# Metabolic Effects of Selective Deletion of Group VIA Phospholipase A_2_ from Macrophages or Pancreatic Islet Beta-Cells

**DOI:** 10.3390/biom10101455

**Published:** 2020-10-17

**Authors:** John Turk, Haowei Song, Mary Wohltmann, Cheryl Frankfater, Xiaoyong Lei, Sasanka Ramanadham

**Affiliations:** 1 Mass Spectrometry Facility, Division of Endocrinology, Metabolism, and Lipid Research, Department of Medicine, Department of Pathology and Immunology, Washington University School of Medicine, St. Louis, MO 63110, USA; songhw@gmail.com (H.S.); wohltmann@wustl.edu (M.W.); c.frankf@wustl.edu (C.F.); 2 Sigma-Aldrich Corporation, St. Louis, MO 63118, USA; 3 Department of Cell, Developmental, and Integrative Biology, Comprehensive Diabetes Center, University of Alabama at Birmingham, Birmingham, AL 35294, USA; xlei@uab.edu

**Keywords:** pancreatic islets, β-cells, insulin secretion, glucose tolerance, insulin resistance, group VIA phospholipase A_2_

## Abstract

To examine the role of group VIA phospholipase A_2_ (iPLA_2_β) in specific cell lineages in insulin secretion and insulin action, we prepared mice with a selective iPLA_2_β deficiency in cells of myelomonocytic lineage, including macrophages (MØ-iPLA_2_β-KO), or in insulin-secreting β-cells (β-Cell-iPLA_2_β-KO), respectively. MØ-iPLA_2_β-KO mice exhibited normal glucose tolerance when fed standard chow and better glucose tolerance than floxed-iPLA_2_β control mice after consuming a high-fat diet (HFD). MØ-iPLA_2_β-KO mice exhibited normal glucose-stimulated insulin secretion (GSIS) in vivo and from isolated islets ex vivo compared to controls. Male MØ-iPLA_2_β-KO mice exhibited enhanced insulin responsivity vs. controls after a prolonged HFD. In contrast, β-cell-iPLA_2_β-KO mice exhibited impaired glucose tolerance when fed standard chow, and glucose tolerance deteriorated further when introduced to a HFD. β-Cell-iPLA_2_β-KO mice exhibited impaired GSIS in vivo and from isolated islets ex vivo vs. controls. β-Cell-iPLA_2_β-KO mice also exhibited an enhanced insulin responsivity compared to controls. These findings suggest that MØ iPLA_2_β participates in HFD-induced deterioration in glucose tolerance and that this mainly reflects an effect on insulin responsivity rather than on insulin secretion. In contrast, β-cell iPLA_2_β plays a role in GSIS and also appears to confer some protection against deterioration in β-cell functions induced by a HFD.

## 1. Introduction

Glycerophospholipids are the most abundant molecular components of biological membrane bilayers and are both critical determinants of membrane structure and the source of signaling molecules produced from their hydrolysis by phospholipase enzymes. Glycerophospholipids consist of a glycerol backbone with a phosphate ester in the *sn*-3 position that may form a phosphodiester linkage to a polar head-group, such as choline, ethanolamine, serine, inositol, or glycerol, inter alia. A fatty acid is esterified to the glycerol backbone in the *sn*-2 position of phospholipids, and in the *sn*-1 position there is an ester, ether, or vinyl ether linkage to a fatty acid, fatty alcohol, or fatty aldehyde residue, respectively. Phospholipase A_2_ (PLA_2_) enzymes hydrolyze the phospholipid *sn*-2 ester bond to yield a free fatty acid and a 2-lysophospholipid as products [1,2]. The PLA_2_ superfamily consists of at least 16 groups of structurally and functionally diverse enzymes that include secreted (sPLA_2_), cytosolic (cPLA_2_), calcium-independent (iPLA_2_), lipoprotein-associated (Lp-PLA_2_), and adipose-PLA_2_ (AdPLA). These enzymes play central roles in cellular lipid metabolism and signaling [1].

The enzyme that is now designated as Group VIA PLA_2_ was the first recognized mammalian member of the Ca^2+^-independent PLA_2_ enzymes [3,4,5], and its abbreviated designation is iPLA_2_β [6,7]. Akin to the plant lipase patatin, iPLA_2_β contains a GXSXG lipase consensus sequence in the enzyme catalytic center, in which the central Ser is a component of a Ser-Asp catalytic dyad [8]. Enzymes with patatin-like phospholipase domains comprise the PNPLA family, and the human genome expresses nine members of this family, of which iPLA_2_β is PNPLA9 [9,10]. The phenotypic properties of experimental mouse models with induced mutations in the genes that encode PNPLA family members and the clinical phenotypes of patients with corresponding mutations indicate that several of these lipases play important metabolic roles in mammalian lipid and energy homeostasis [9,10].

Global iPLA_2_β-null mice produced by homologous recombination exhibit several phenotypic abnormalities, including greatly impaired male fertility [11] and the development of a neurodegenerative condition [12] that is similar to the human genetic disease Infantile Neuroaxonal Dystrophy (INAD), which arises from mutations in the Group VIA PLA_2_ gene [13,14]. Consistent with previous evidence that iPLA_2_β participates in signaling events leading to glucose-stimulated insulin secretion (GSIS) from pancreatic islet β-cells [15,16,17,18,19,20,21,22], islets isolated from male [23] or female [24] iPLA_2_β-null mice exhibit impaired GSIS ex vivo, and male iPLA_2_β-null mice exhibit impaired glucose tolerance in vivo [23]. In contrast, female iPLA_2_β-null mice exhibit normal glucose tolerance in the unstressed state [24] but develop a more severe glucose intolerance than wild-type littermates after exposure to the β-cell toxin streptozotocin or after the introduction of a high-fat diet (HFD) [24]. Surprisingly, female iPLA_2_β-null mice experienced less deterioration in insulin sensitivity than did wild-type littermates after being introduced to a HFD, although pancreatic islets isolated from HFD-fed female iPLA_2_β-null mice exhibited a much more severe impairment of GSIS than did islets isolated from HFD-fed wild-type littermates [24].

This discordance of the effects of a HFD on insulin secretion and insulin sensitivity in global iPLA_2_β-null mice suggests that iPLA_2_β plays distinct roles in the molecular mechanisms underlying insulin secretion and insulin action and in the impact of a HFD on these processes. Evidence indicates that iPLA_2_β amplifies glucose-induced Ca^2+^ entry into β-cells [23], suggesting that this is one component of the mechanism(s) through which iPLA_2_β participates in signaling events underlying GSIS, and iPLA_2_β also participates in the repair of β-cell mitochondrial membranes that are oxidized upon exposure to high concentrations of palmitic acid [25]. This might represent a mechanism whereby iPLA_2_β mitigates β-cell injury in HFD-fed mice and accounts for the fact that loss of iPLA_2_β results in a greater impairment of insulin secretion in HFD-fed iPLA_2_β-null mice compared to HFD-fed wild-type littermates [24].

Macrophages and their precursor monocytes also express iPLA_2_β [26,27,28,29], and its expression level affects the macrophage phenotype [30]. Migration of monocytes into extravascular sites, including adipose tissue, and their differentiation into macrophages that elaborate cytokines, including TNFα and IL-6, which impair insulin sensitivity, are thought to represent a critical series of events in the development of diet-induced insulin resistance in diabetes and obesity and to involve tissue elaboration of the cytokine Monocyte Chemoattractant-1 (MCP-1) and its interaction with the monocyte MCP-1 receptor CCR2 [31,32,33,34,35,36]. Genetic and pharmacologic evidence indicates that the monocyte chemotactic response to MCP-1 requires the action of iPLA_2_β [37,38,39] to generate the lipid mediator 2-lysophosphatidic acid (LPA) [29,37,39]. These observations suggest the possibility that the relative insensitivity of iPLA_2_β-null mice to high-fat diet (HFD)-induced insulin resistance might reflect the failure of iPLA_2_β-null monocytes to migrate into adipose tissue and other extravascular sites in response to HFD-induced tissue elaboration of MCP-1.

A plausible hypothesis is thus that the net metabolic effects of global deletion of iPLA_2_β might reflect opposing effects on β-cells and monocyte-macrophages. A loss of iPLA_2_β in β-cells would result in impaired insulin secretion and increased sensitivity to lipid-induced injury, but loss of iPLA_2_β in macrophages might provide relative protection against the HFD-induced deterioration of insulin sensitivity because of an impaired migration of monocytes into extravascular tissues, differentiation into macrophages, and elaboration of cytokines that result in insulin resistance. To test this hypothesis, we generated mice with floxed-iPLA_2_β alleles and mated them with mice that express Cre recombinase under control of LysM or RIP2 promoters to produce mice with selective iPLA_2_β deficiency in macrophages (MØ-iPLA_2_β-KO) or insulin-secreting β-cells (β-cell-iPLA_2_β-KO), respectively. The metabolic phenotypes of these mice were then characterized and are described in this report.

## 2. Materials and Methods

### 2.1. Materials

Enhanced chemiluminescence reagents were obtained from Amersham Biosciences (Piscataway, NJ, USA); SDS-PAGE supplies from Bio-Rad (Richmond, CA, USA); ATP, common reagents, and salts from Sigma (St. Louis, MO, USA); culture media, penicillin, streptomycin, Hanks’ balanced salt solution, L-glutamine, agarose, and RT-PCR reagents from Invitrogen (Carlsbad, CA, USA); fetal bovine serum from Hyclone (Logan, UT); Pentex bovine serum albumin (BSA, fatty acid-free, fraction V) from ICN Biomedical (Aurora, OH, USA); forskolin from Calbiochem (La Jolla, CA, USA). Krebs–Ringer bicarbonate (KRB) buffer contained (in mM) 25 HEPES (pH 7.4), 115 NaCl, 24 NaHCO_3_, 5 KCl, 1 MgCl_2_, and 2.5 CaCl_2_.

### 2.2. Preparation of Mice with Selective Deletion of iPLA_2_β from Restricted Cell Lineages

**Figure 1:**
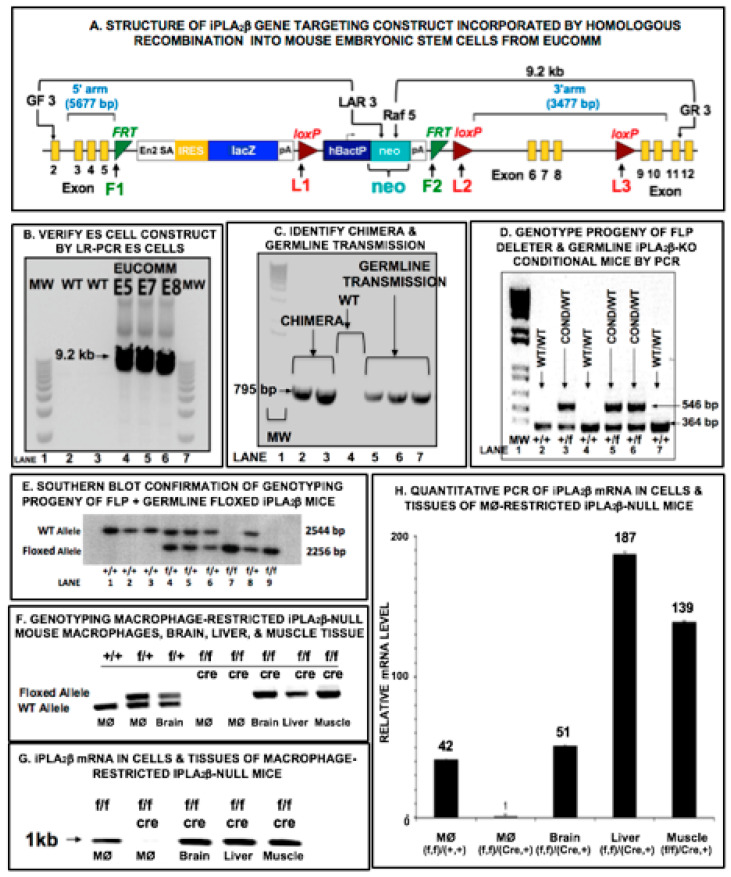
Cre-Lox Preparation of Mutant Mice with Tissue-Restricted Deletion of iPLA_2_β. (**A**) is a schematic illustration of the structure of the gene-targeting construct that was incorporated by homologous recombination into mouse embryonic stem (ES) cells from European Conditional Mouse Mutagenesis (EUCOMM). (**B**) demonstrates correct integration of the 5′ and 3′ arms of the construct in the ES cell lines by long-range PCR using primer sets that recognize sequences external to the construct and within the neo cassette, respectively, to yield a 9.2 kb product. (**C**) illustrates that injection of the ES cells into blastocysts that were then implanted into pseudo-pregnant female mice resulted in production of chimeric mice with germline transmission of the targeted gene to yield heterozygotes for the wild-type (WT) and EUCOMM iPLA_2_β alleles. (**D**) illustrates that mating of these heterozygotes with FLP deleter mice resulted in removal of the region between F1 and F2 sites in the targeting construct that contained the neo cassette to yield an iPLA_2_β allele that contained Lox2 and Lox3 sites flanking iPLA_2_β exons 6–8 and that this allele is distinguishable from the WT allele upon PCR analyses. (**E**) is a Southern blot of gene fragments after Bam H1 endonuclease digestion and illustrates production of fragments characteristic of the WT and conditional alleles that establish the genotype of individual mice. WT and conditional iPLA_2_β heterozygotes were mated to yield conditional allele homozygotes, heterozygotes, and WT allele homozygotes in a 1:2:1 ratio. Those mice were then mated with mice that express Cre recombinase under control of LysM or RIP2 promoters to drive Cre expression selectively in macrophages or pancreatic islet β-cells, respectively. (**F**) illustrates that with conditional allele homozygotes these matings result in selective iPLA_2_β deletion in only the targeted cell line and not in other tissues. (**G**,**H**) illustrate that iPLA_2_β mRNA is not produced in the Cre-expressing cell type in mice that are iPLA_2_β conditional allele homozygotes but that iPLA_2_β mRNA is produced by non-targeted tissues.

The Washington University Animal Studies Committee approved all animal studies. Mice with floxed-iPLA_2_β alleles were prepared and mated with mice expressing Cre recombinase under control of cell type-restricted promoters to generate conditionally iPLA_2_β-deficient mice. European Conditional Mouse Mutagenesis (EUCOMM) embryonic stem (ES) cells with an iPLA_2_β-targeting construct incorporated by homologous recombination [40] were purchased (Figure 1A). In this construct, LoxP sites L2 and L3 were recognized by Cre recombinase [41,42] flank critical iPLA_2_β gene exons 6–8 (Figure 1A). Removing this “floxed” segment results in a truncated mRNA species eliminated by nonsense mediated decay. The 5′ fragment with the neo cassette is flanked by FRT (flippase recognition target) sites F1 and F2 (Figure 1A) that are recognized by FLP (flippase) recombinase [43,44]. Correct integration of 5′ and 3′ arms was confirmed by PCR (Figure 1B) using primer sets that recognize sequences external to the construct and within the neo cassette, respectively. Primer set Raf 5 and GR3 for the 3′ arm yielded a 9.2 kb product (Figure 1A). Two clones (G05, G07) with normal karyotypes were injected into blastocysts that were then implanted into pseudo-pregnant females. Both yielded chimeric mice that transmitted the targeted allele in the germ line to yield heterozygotes for wild-type (WT) and EUCOMM iPLA_2_β alleles [45], as verified by PCR with primers within the neo cassette and the intron between L2 and exon 6, respectively, that yield a 795 bp product (Figure 1C).

These mice were mated with FLP deleter mice to excise the F1-F2 region that contained the neo cassette [43,44,45] to yield a conditional iPLA_2_β allele in which loxP sites L2 and L3 flank exons 6–8 (Figure 1A). Progeny included heterozygotes for conditional and WT iPLA_2_β alleles, as verified by PCR genotyping with primers in introns between exon 5 and F2 and between L2 and exon 6, respectively, which yielded products of 546 and 364 bp for conditional and WT alleles, respectively (Figure 1D). Genotypes were confirmed by Southern blotting after digestion with restriction endonuclease Bam H1, which cleaves at sites B1, B2 (conditional allele), and B3 (WT allele) to yield fragments of 2544 and 2256 bp for WT and conditional alleles (Figure 1E), respectively, recognized by a probe to the 3′ end of exon 4 and the following intron. Heterozygous (conditional/WT) mice were mated with mice that express Cre recombinase under control of RIP2 or LysM promoters to direct Cre expression in β-cells or macrophages [46,47,48,49,50,51,52,53], respectively. FLP-negative offspring were mated with Cre mice to produce mice homozygous for iPLA_2_β conditional alleles that express Cre recombinase (Figure 1F) and produce no iPLA_2_β mRNA in macrophages but do so in other tissues (Figure 1G,H).

Mice were housed in a specific pathogen-free barrier facility with unrestricted access to water and standard mouse chow containing 6% fat. For mice with a β-cell-specific inactivation of iPLA_2_β (β-iPLA_2_β-KO), RIP2-Cre mice (The Jackson Laboratory, number 003573) were crossed with mice carrying iPLA_2_β alleles with exons 6–8 flanked by loxP recombination sites (iPLA_2_β^lox/lox^) [46]. First, generation animals hemizygous for the RIP-Cre gene and bearing one “floxed” LPL allele (iPLA_2_β^lox/wt^ Cre^+^) were crossed with iPLA_2_β^lox/lox^ animals to generate β-cell iPLA_2_β-deficient (iPLA_2_β^lox/lox^ Cre^+^) and β-cell iPLA_2_β wild-type (iPLA_2_β^lox/lox^ Cre^-^) littermates that were at least N5 in the C57BL/6 background with a conditional deletion of iPLA_2_β in β-cells. The following primers were used to document iPLA_2_β gene rearrangement: primer A, 5′-CCCAGCTCTGTGTCTTAGTATG-3′; primer B, 5′-TTCTTGGCCCAATGGAGTG-3′. Amplification of WT DNA yields a product of 673 bp; amplification of non-rearranged floxed DNA allele yields a product of 855 bp, whereas the amplification of appropriately rearranged DNA will not show a band since the exon 5 is deleted. For mice with a myelomonocytic cell-specific inactivation of iPLA_2_β (MØ-iPLA_2_β-KO), mice with loxP-flanked iPLA_2_β alleles were mated with lysozyme M-Cre mice [51] and crossbred to yield iPLA_2_β knock-out in macrophage (MØ-iPLA_2_β-KO) mice that were at least N5 in the C57BL/6 background with conditional deletion of iPLA_2_β in the myelomonocytic lineage. Mice were genotyped using iPLA_2_β- and Cre-specific primer sets [54], weaned to chow providing 6% calories as fat, and subsequently fed a high-fat diet (HFD), as described below.

### 2.3. Analyses of iPLA_2_β mRNA in Mouse Tissues

Northern blots of iPLA_2_β mRNA were performed as described in [11]. For RT-PCR, total RNA was isolated with an RNeasy kit (Qiagen Inc.). A SuperScript First Strand Synthesis System (Invitrogen) was used to synthesize cDNA in 20 µL reactions that contained DNase I-treated total RNA (2 µg). The cDNA product was treated (20 min, 37 °C) with RNase H (2 units, Invitrogen), and was heat inactivated (70 °C for 15 min). A reaction without reverse transcriptase was performed to verify the absence of genomic DNA. The PCR performed with the pair of primers 1 and 2 was designed to amplify a fragment that spans the neomycin cassette insertion site. The PCR performed with the pair of primers 3 and 2 was designed to amplify a fragment downstream from the neomycin cassette insertion site. The sequence of primer 1 is tgtgacgtggacagcactagc; that of primer 2 is ccccagagaaacgactatgga; that of primer 3 is tatgcgtggtgtgtacttccg.

### 2.4. High-Fat Dietary Intervention Studies

Mice were housed in a pathogen-free barrier facility with unrestricted access to water and standard mouse chow (Purina Mills Rodent Chow 5053) with a caloric content of 13.025% fat, 62.144% carbohydrate, and 24.651% protein. For dietary intervention studies, mice were fed standard chow until 8 weeks of age and thereafter were randomized into groups that were fed either standard chow or a HFD continuously until they reached the age of three or six months, respectively, as described in [55]. The HFD (Harlan Teklad catalog TD88137) had a caloric content of 42% fat, 42.7% carbohydrate, and 15.2% protein.

### 2.5. Blood Glucose and Insulin Concentrations

As described previously [56], blood samples were obtained from the lateral saphenous vein in heparinized capillary tubes, and glucose concentrations were measured in whole blood with a blood-glucose monitor (Becton Dickenson) or an Ascensia ELITE XL blood-glucose meter. Plasma was prepared from heparinized blood by centrifugation, and insulin levels were determined in aliquots (5 µL) with a rat insulin ELISA kit (Crystal Chem). Fasting blood samples were obtained after an overnight fast, and fed blood samples were obtained between 9:00 and 10:00 a.m.

### 2.6. Glucose and Insulin Tolerance Tests

As described in [23], intraperitoneal glucose tolerance tests (IPGTTs) were performed on mice that fasted overnight from which a baseline blood sample was obtained, followed by intraperitoneal injection of D-glucose (2 mg/g body weight) and collection of blood for measurement of glucose level after 30, 60, and 120 min. Insulin tolerance tests were performed in mice with free access to water and chow that received an intraperitoneal injection (0.75 U/kg body weight) of human regular insulin (Lilly, Indianapolis, IN, USA), followed by collection of blood after 30, 60, and 120 min for glucose level determinations [24,26].

### 2.7. Area Under the Curve (AUC) Calculations for Glucose Tolerance Tests (GTTs)

As described previously [23,24,57], the AUC for the GTT curves was calculated by the method of Sakaguchi et al. [58], where the blood glucose concentration at t = x min is designated G(x):

AUC = [0.25 × G(0)] + [0.5 × G(30)] + [0.75 × G(60)] + [0.5 × G(120)](1)

### 2.8. Insulin Secretion In Vivo

Mice were fasted overnight, and baseline blood samples were obtained from the saphenous vein, followed by intraperitoneal injection of D-glucose (3 mg/kg body weight), and a blood sample was obtained 30 min thereafter for the measurement of plasma insulin levels, as described in [57].

### 2.9. Pancreatic Islet Isolation

Islets were isolated from pancreata removed from mice by collagenase digestion after mincing, followed by Ficoll step density gradient separation, and manual selection under stereomicroscopic visualization to exclude contaminating tissues [46,59]. 

### 2.10. Insulin Secretion Ex Vivo from Isolated Pancreatic Islets in Static Incubations

Islets were rinsed with KRB medium containing 3 mM glucose and 0.1% bovine serum albumin and placed in silanized tubes (12 × 75 mm) in the same buffer, through which 95% air/5% CO_2_ was bubbled before the incubation. The tubes were capped and incubated (37 °C, 30 min) in a shaking water bath, as described in [24,46,59]. The buffer was then replaced with KRB medium containing 1, 11, or 20 mM glucose and 0.1% BSA without or with forskolin (2.5 µM), and the samples were incubated for 30 min. Insulin secreted into the medium was measured, as described in [46,57].

### 2.11. Other Analytical Procedures

Serum glucose was measured using reagents from Sigma, and serum insulin was measured by an enzyme-linked immunosorbent assay (Crystal Chem. Inc., Downer’s Grove, IL, USA), as described in [46].

### 2.12. Statistical Methods

Results are presented as mean ± SEM. Data were evaluated by an unpaired, two-tailed Student’s *t* test or by an analysis of variance with appropriate post-hoc tests. Significance levels are described in the figure legends.

## 3. Results

### 3.1. Mouse Genotype Characterization

As described in the experimental procedures and illustrated in Figure 1***,*** mice homozygous for a floxed-iPLA_2_β allele were prepared and mated with mice that express Cre recombinase in a restricted set of tissues to produce offspring with conditional iPLA_2_β gene deletions. Such mice fail to express iPLA_2_β in tissues that express Cre because the floxed gene is excised by the action of the recombinase, but those mice do express iPLA_2_β in all other tissues. Two breeding lines of mice with a tissue-selective expression of Cre were used, one of which expresses Cre under control of the Rat Insulin Promoter (RIP) which is active in insulin-secreting pancreatic islet β-cells and in a limited number of other cells but not in the vast majority of cells [46,47,48,49,50]. When mated with mice homozygous for a floxed-iPLA_2_β allele, some progeny, which are identified by genotyping, fail to express iPLA_2_β in β-cells, and their genotype is designated β-cell-iPLA_2_ β-KO. The second breeding line expresses Cre under control of the Lysozyme-M (Lys) promoter that is active in myelomonocytic lineage cells, including monocyte/macrophages [51,52]. When mated with mice homozygous for a floxed-iPLA_2_β allele, some progeny, again identified by genotyping, fail to express iPLA_2_β in monocyte/macrophages (MØ), and their genotype is designated MØ-iPLA_2_β-KO. β-Cell-iPLA_2_β-KO mice are thus selectively deficient in iPLA_2_β in β-cells, and MØ-iPLA_2_β-KO mice are selectively deficient in iPLA_2_β in monocyte/macrophages. Mice homozygous for a floxed-iPLA_2_β allele that do not express Cre are designated “Floxed-iPLA_2_β” and serve as controls when examining the metabolic behavior of the conditional iPLA_2_β-KO mice.

### 3.2. Glucose Tolerance Tests

**Figure 2:**
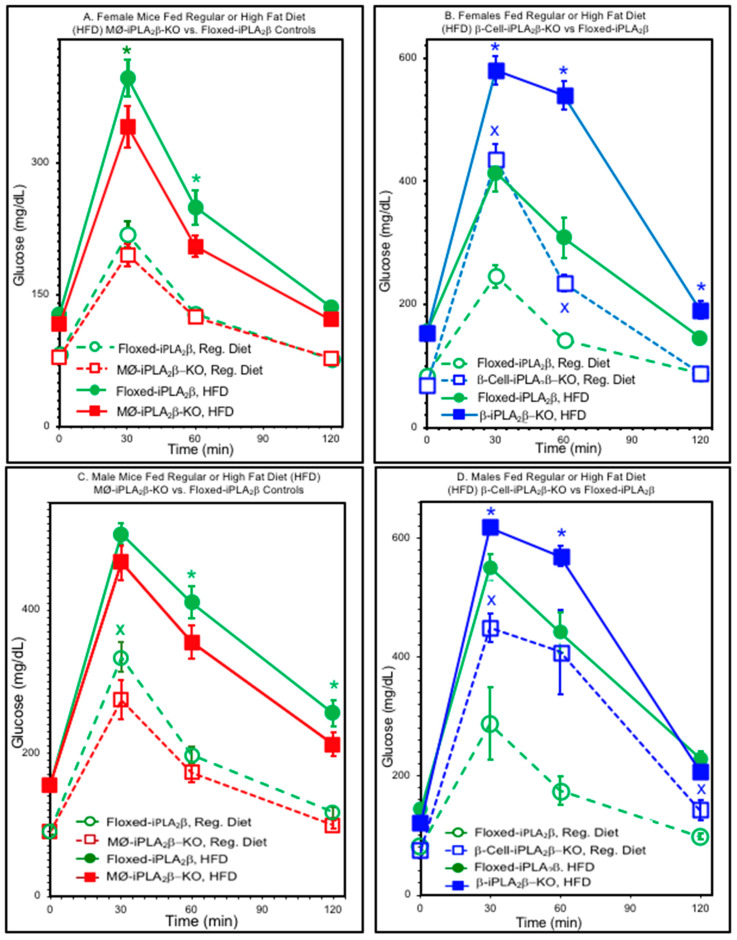
Glucose tolerance tests for iPLA_2_β conditional knockout mice and floxed-iPLA_2_β controls. D-glucose (2 mg/g body weight) was administered by intraperitoneal injection to female (**A,B**) or male (**C**,**D**) floxed-iPLA_2_β control mice (circles), MØ- iPLA_2_β-KO mice (**A**,**C**, squares), or β-cell-iPLA_2_β-KO mice (**B**,**D**, squares) 6 months of age that had been fed a regular diet (open symbols) or high-fat diet (HFD, closed symbols) after the age of 8 weeks, and blood was collected at baseline and at 30, 60, and 120 min after glucose administration to measure blood glucose concentration. Values are displayed as means ± SEM (n = 6 to 24, as specified by condition in Table S1). An asterisk (*) denotes *p* < 0.05 for comparisons between genotypes. The symbol x denotes *p* < 0.05 for the comparison between diets.

Glucose tolerance tests (GTTs) performed with female mice 6 months of age of various genotypes after consuming food from a regular diet (RD) or high-fat diet (HFD) are illustrated in Figure 2, in which the blood glucose concentration is plotted as a function of time after an intraperitoneal administration of glucose.

Figure 2A shows that for floxed-iPLA_2_β control mice, glucose tolerance deteriorates significantly in HFD-fed mice compared to RD-fed mice. This effect of diet was also observed in MØ-iPLA_2_β-KO mice, but the peak glucose concentration and the area under the curve (AUC) of the GTTs were both significantly lower for MØ-iPLA_2_ β-KO mice than for floxed-iPLA_2_β controls, suggesting that MØ-selective iPLA_2_β deficiency confers some protection against diet-induced glucose intolerance.

Figure 2B illustrates that GTTs performed with β-cell-iPLA_2_ β-KO mice compared to floxed-iPLA_2_β controls. Again, there is HFD-induced deterioration in GTTs compared to RD-fed mice for the floxed-iPLA_2_β control mice. This dietary effect was also observed in β-cell-iPLA_2_β-KO mice. In contrast to MØ-iPLA_2_β-KO mice, the peak glucose concentration and the Area Under the Curve (AUC) of the GTT were both significantly higher in β-cell-iPLA_2_β-KO mice than in floxed-iPLA_2_β controls, suggesting that β-cell-selective iPLA_2_β deficiency exacerbates diet-induced glucose intolerance.

**Figure 3:**
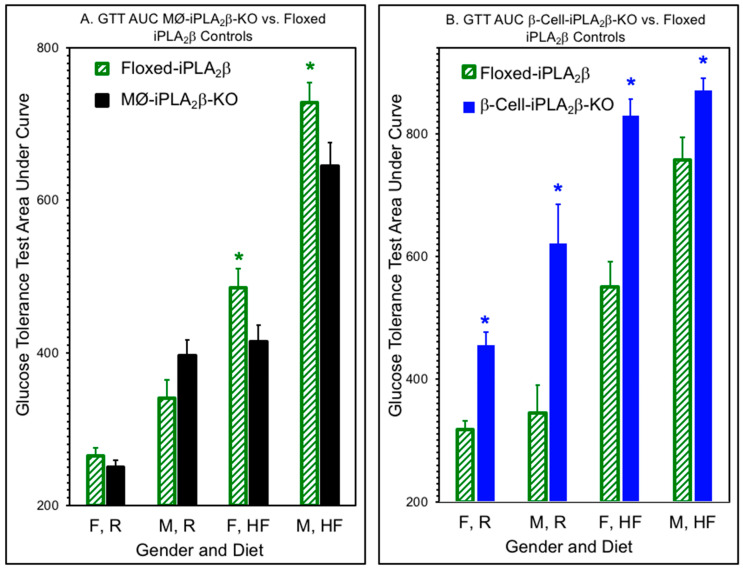
Areas under the curve for glucose tolerance tests for iPLA_2_β conditional knockout mice and floxed-iPLA_2_β controls. Glucose tolerance tests (GTTs) were performed as in Figure 2 for male (M) or female (F) MØ-iPLA_2_β-KO mice (**A**), β-cell-iPLA_2_β-KO mice (**B**), or floxed-iPLA_2_β control mice 6 months of age that had been fed a regular (R) or high-fat (HF) diet, and the Areas Under the Curves (AUCs) were calculated from the measured glucose concentration values by the trapezoidal method of Sakaguchi et al. [58]. Values are displayed as means ± SEM (n = 6 to 24, as specified by condition in Table S1). An asterisk (*) denotes *p* < 0.05 for comparisons between genotypes.

Similar effects of genotype and diet were observed in male mice 6 months of age, as illustrated in Figure 3, in which the AUC of the GTT is plotted for female (F) or male (M) mice fed a regular (R) or high-fat (HF) diet. Figure 3A shows that for a given diet, males exhibit higher GTT AUC values than females and that a HFD causes deterioration in glucose tolerance, as reflected by a higher AUC, compared to RD-fed mice. For both males and females, the diet-induced rise in GTT AUC was significantly lower for MØ-iPLA_2_β-KO mice than for floxed-iPLA_2_β controls. In contrast, the diet-induced rise in GTT was significantly higher for β-KO (RIP) mice than for floxed-iPLA_2_β controls for both males and females.

Together, Figure 2 and Figure 3 demonstrate that glucose tolerance is affected by diet, gender, and genotype, with a higher GTT AUC for males compared to females and for HFD-fed compared to RD-fed mice. Compared to flox mice, MØ-iPLA_2_β-KO mice exhibit a significantly lower HFD-diet induced deterioration of GTT than floxed-iPLA_2_β controls. In contrast, β-cell-iPLA_2_β-KO mice exhibit significantly poorer glucose tolerance, as reflected by a higher GTT AUC than floxed-iPLA_2_β controls after the introduction of an RD, and HFD-induced deterioration of glucose tolerance is greater for β-cell-iPLA_2_β-KO mice than for floxed-iPLA_2_β controls.

Table S1 illustrates an effect of age on glucose tolerance. Metabolic abnormalities that have developed in mice aged 6 months were found to be nascent but attenuated in mice aged 3 months. For a given condition, GTT AUC is lower for mice aged 3 months than for mice aged 6 months. A HFD also induces deterioration in glucose tolerance for both female and male mice at an age of 3 months, although the effect is smaller than with mice aged 6 months. For female mice aged 3 months, the GTT AUC was significantly lower for MØ-iPLA_2_β-KO mice than for floxed-iPLA_2_β control mice after RD-consumption, and the GTT AUC was significantly higher for β-cell-iPLA_2_ β-KO mice than for floxed-iPLA_2_β mice after RD-consumption. No other comparisons were statistically significant for female or male mice aged 3 months, although there was a strong trend for higher GTT AUC for HFD-fed β-Cell-iPLA_2_β-KO mice than for floxed-iPLA_2_β controls and a trend for higher GTT AUC for RD-fed male β-cell-iPLA_2_β-KO mice than for floxed-iPLA_2_β controls. Weaker trends were observed for the GTT AUC to be lower in HFD-fed female MØ-iPLA_2_β-KO mice than for floxed-iPLA_2_β mice and for the GTT AUC to be higher in HFD-fed male β-cell-iPLA_2_β-KO mice than for floxed-iPLA_2_β controls.

### 3.3. Ex Vivo Insulin Secretion from Isolated Pancreatic Islets

**Figure 4:**
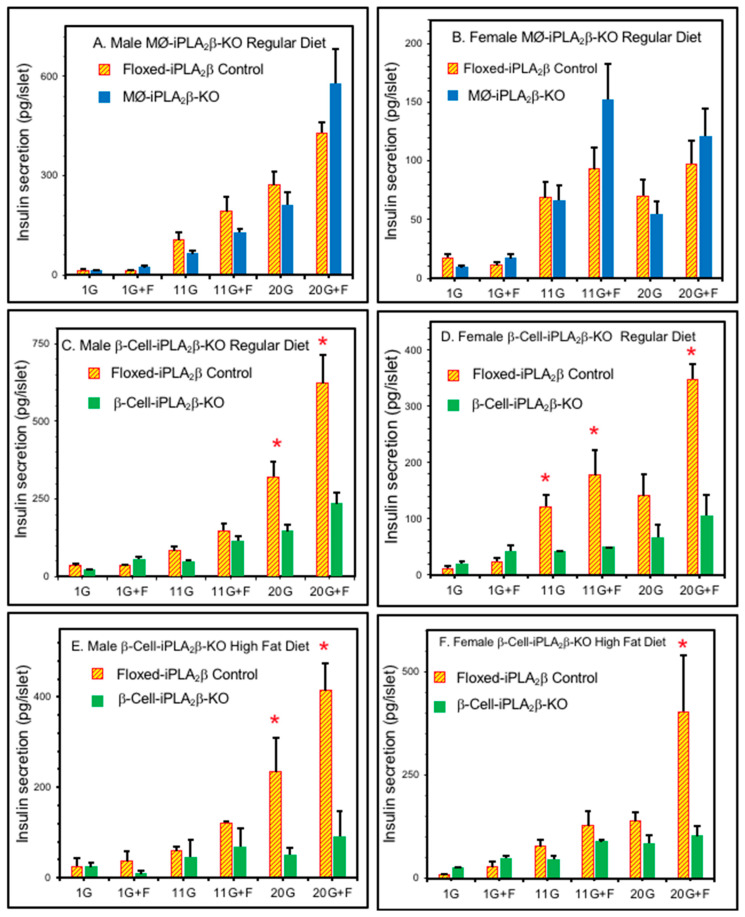
Insulin secretion by pancreatic islets isolated from iPLA_2_β conditional knockout mice and floxed-iPLA_2_β controls. Insulin secretion was stimulated by d-glucose and forskolin from pancreatic islets isolated from male (**A**,**C**,**E**) or female (**B**,**D**,**F**) MØ-iPLA_2_β-KO mice (**A**,**B**, solid bars), β-cell-iPLA_2_β-KO mice (**C–F**, solid bars), or floxed-iPLA_2_β control mice (**A**–**F**, cross-hatched bars) 6 months of age that had been fed a RD (**A–D**) or a HFD (**E**,**F**). Incubations (30 islets per condition, 30 min, 37 °C) were performed in buffer containing 1, 11, or 20 mM d-glucose without or with 2.5 µM forskolin, and an aliquot of medium was then removed for measurement of insulin. Mean values ± SEM are displayed (n = 4, in triplicate). An asterisk (*) denotes *p* < 0.05 for the comparison between genotypes.

The magnitude of GSIS from pancreatic islet β-cells has an important influence on glucose tolerance, and the secretory behavior of pancreatic islets isolated from RD-fed or HFD-fed mice of various genotypes was therefore examined ex vivo, as illustrated in Figure 4. Islets isolated from both male and female RD-fed floxed-iPLA_2_β control mice exhibited insulin secretion that increased with medium glucose concentration over a range from 1 to 20 mM, and this response was amplified in the presence of the adenylyl cyclase activator, forskolin (Figure 4A–D), as observed in previous studies [22,23,24]. Insulin secretory responses from RD-fed MØ-iPLA_2_β-KO male or female mice were not statistically different from those for floxed-iPLA_2_β controls (Figure 4A,B). In contrast, insulin secretory responses from RD-fed β-cell-iPLA_2_β-KO male or female mice were significantly lower from those for floxed-iPLA_2_β controls (Figure 4C,D), and this was also observed in islets from HFD-fed β-cell-iPLA_2_β-KO male and female mice (Figure 4D,E), which exhibited even lower insulin secretory responses relative to floxed-iPLA_2_β controls than did islets from RD-fed mice. These observations indicate that selective iPLA_2_β deficiency in β-cells results in impaired insulin secretion from pancreatic islets but that selective iPLA_2_β deficiency in MØ does not. The impaired islet insulin secretion of β-cell-iPLA_2_β-KO mice in Figure 4C–F is thus likely to contribute to the impaired glucose tolerance in these mice (Figure 2B and Figure 3B).

### 3.4. In Vivo Insulin Secretion in Mice After Intraperitoneal Glucose Administration

**Figure 5:**
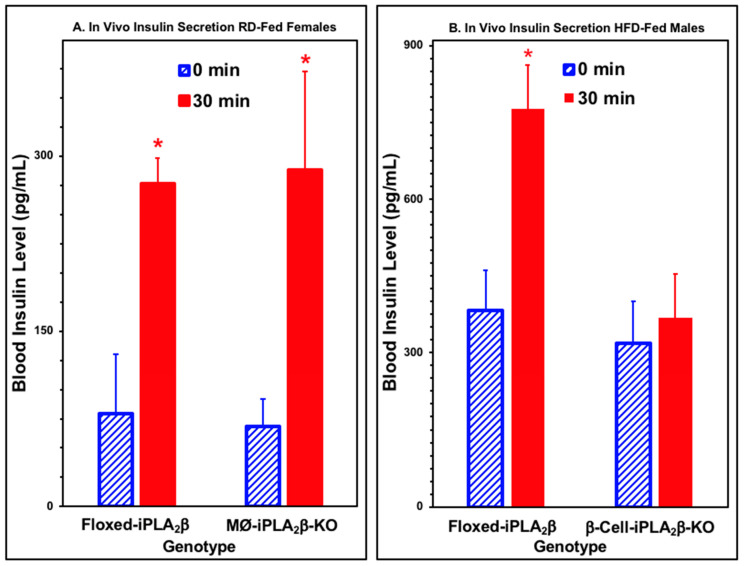
In vivo insulin secretion for iPLA_2_β conditional knockout mice and floxed-iPLA_2_β control mice fed a regular (RD) or high-fat diet (HFD). After an overnight fast, baseline blood samples were obtained from the saphenous vein of male (**A**) or female (**B**) MØ-iPLA_2_β-KO mice, β-cell-iPLA_2_β-KO mice, or floxed-iPLA_2_β control mice 6 months of age that had been fed a regular diet (RD, **A**) or high-fat diet (HFD, **B**). d-glucose (3 mg/kg body weight) was administered by intraperitoneal injection, and a blood sample was obtained 30 min thereafter. The insulin contents of the baseline and 30 min samples were then measured by enzyme-linked immunosorbent assay, as described [23,24,57]. Displayed values represent mean ± SEM. An asterisk (*) denotes *p* < 0.05 for the comparison between the time 0 and 30 min values (n = 5 to 28).

To determine whether insulin secretion in vivo would reflect the effects of genotype on insulin secretion ex vivo from isolated pancreatic islets, we determined the increment in blood insulin concentration that occurred 30 min after an intraperitoneal administration of glucose to mice of various genotypes, gender, and dietary history (Figure 5). Both RD-fed male and HFD-fed female floxed-iPLA_2_β control mice exhibited a significant increment in blood insulin concentrations after an intraperitoneal administration of glucose (Figure 5A,B and Figure S1). This was also true for MØ-iPLA_2_β-KO mice, and the magnitude of the rise in blood insulin level was similar and not significantly different when comparing MØ-iPLA_2_β-KO mice to floxed-iPLA_2_β controls. This is consistent with the observation that insulin secretion from pancreatic islets isolated from MØ-iPLA_2_β-KO mice is not impaired compared to floxed-iPLA_2_β controls (Figure 4).

In contrast, neither RD-fed female or HFD-fed male β-cell-iPLA_2_β-KO mice exhibited a significant increase in blood insulin levels 30 min after intraperitoneal glucose administration (Figure 5A,B and Figure S1). This is consistent with the impairment of ex vivo glucose-dependent insulin secretion that was observed with pancreatic islets isolated from β-cell-iPLA_2_β-KO mice relative to floxed-iPLA_2_β controls (Figure 4), suggesting that inadequate insulin secretion contributes to the substantially impaired glucose tolerance observed with β-cell-iPLA_2_β-KO mice (Figure 2 and Figure 3).

### 3.5. Insulin Tolerance Tests

**Figure 6:**
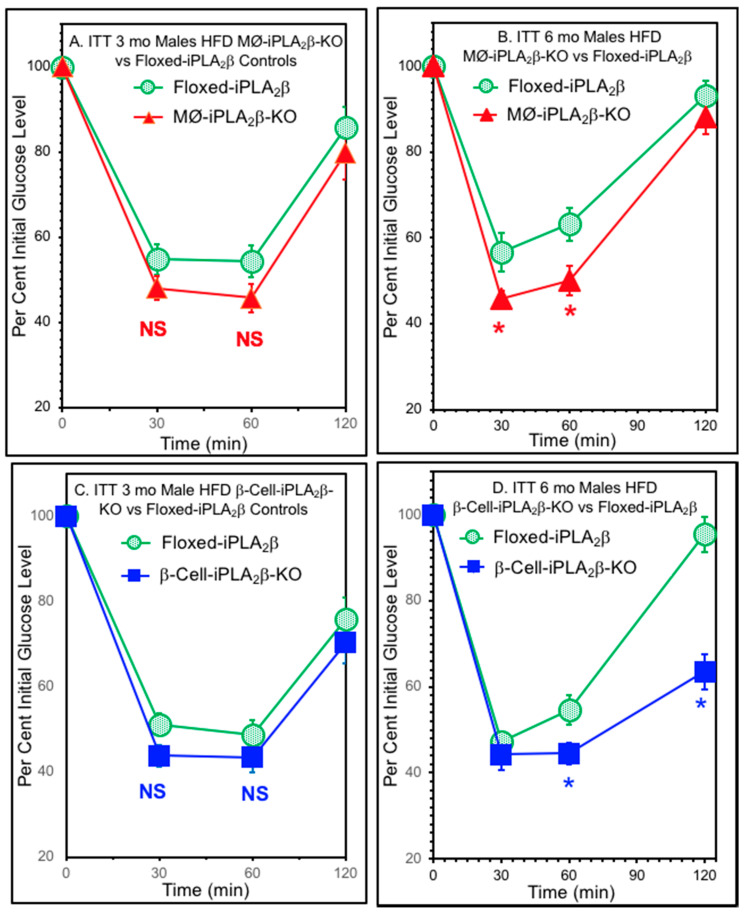
Insulin tolerance tests of 3- or 6-month-old male conditional iPLA_2_β-knockout mice and floxed-iPLA_2_β controls fed a regular or high-fat diet. Male floxed-iPLA_2_β control mice (circles), MØ-iPLA_2_β-KO mice (triangles), or β-cell-iPLA_2_β-KO mice (squares) mice were fed a regular diet after weaning until they were 6 weeks of age and were then fed a HFD until aged 3 months (**A,C**) or 6 months (**B,D**). Insulin tolerance tests were then performed in mice with free access to water and chow until human regular insulin (0.75 U/kg body weight; Lilly, Indianapolis, IN) was administered by intraperitoneal injection. Blood specimens were collected at 0, 30, 60, and 120 min thereafter for glucose concentration measurements, which were expressed as a percentage of the time zero blood glucose concentration, as described in [23,24,57]. Displayed values represent mean ± SEM (n = 13 to 16 (**A**), n = 15 to 18 (**B**), n = 22 to 26 (**C**), and n = 21 to 22 (**D**). An asterisk (*) denotes *p* < 0.05 for the comparison between genotypes.

In addition to the magnitude of GSIS from pancreatic islets, the responsivity of peripheral tissues, including skeletal muscle, to insulin is an important determinant of glucose tolerance. To evaluate insulin responsivity of mice of various genders, genotypes, and dietary history, insulin tolerance tests (ITTs) were performed by measuring the blood glucose concentrations at various times after an intraperitoneal injection of a fixed dose of insulin, as illustrated in Figure 6.

For female mice aged 3 months either RD- or HFD-fed, the ITT curves for neither MØ-iPLA_2_β-KO nor β-cell-iPLA_2_β-KO conditional knockout mice were statistically distinguishable from those for floxed-iPLA_2_β control mice. This was also true for 3-month-old male MØ-iPLA_2_β-KO conditional knockout mice and for HFD-fed 3-month-old male β-cell-iPLA_2_β-KO conditional knockout mice compared to floxed-iPLA_2_β control mice. For 3-month-old male β-cell-iPLA_2_β-KO conditional knockout mice fed a RD, the ITT curves showed a slightly but significantly superior insulin sensitivity compared to floxed-iPLA_2_β control mice (not shown), and this difference was magnified further at age 6 months, as described below.

As illustrated in Figure 6, at 3 months of age the ITTs of male HFD-fed floxed-iPLA_2_β control mice and MØ-iPLA_2_β-KO mice (Figure 6A) did not differ significantly, and this was also true for β-cell-iPLA_2_β-KO mice compared to floxed-iPLA_2_β control mice aged 3 months (Figure 6C). The deterioration of insulin sensitivity with age occurs, and by 6 months of age, a statistically significant difference between the ITTs for HFD-fed male MØ-KO and floxed-iPLA_2_β control mice had developed (Figure 6B). The 6-month-old HFD-fed MØ-iPLA_2_β-KO mice achieved significantly lower blood glucose levels than floxed-iPLA_2_β control mice at 30 and 60 min after insulin administration (Figure 6B). This is consistent with the hypothesis that motivated the preparation of the MØ-iPLA_2_β-KO mice—that HFD feeding would induce a lower deterioration in glucose tolerance and insulin sensitivity in MØ-iPLA_2_β-KO mice compared to floxed-iPLA_2_β control mice, possibly because of impaired migration of monocytes into peripheral tissues where their differentiation into cytokine-producing macrophages ordinarily contributes to insulin resistance. Similar to the phenomenon described above for 3-month-old male β-cell-iPLA_2_β-KO conditional knockout RD-fed mice, at 6 months of age HFD-fed β-cell-iPLA_2_β-KO mice were also significantly more responsive to insulin than floxed-iPLA_2_β control mice (Figure 6D), raising the unanticipated possibility that β-cell products might also contribute to the development of HFD-induced insulin resistance.

A similar phenomenon was observed in 6-month-old HFD-fed female β-cell-iPLA_2_β-KO mice, which exhibited superior insulin sensitivity compared to floxed-β-cell-iPLA_2_β-KO control mice, and this magnified a smaller but similar trend observed with 6-month-old female β-cell-iPLA_2_β-KO mice fed regular chow (Figure S2A,B). For 6-month-old female MØ-iPLA_2_β-KO mice, the ITT curves did not differ significantly from those of floxed-iPLA_2_β control mice fed either a RD or a high-fat diet HFD (Figure S2C,D). Curiously, 6-month-old male MØ-iPLA_2_β-KO RD-fed mice exhibited lower insulin sensitivity compared to floxed-iPLA_2_β control mice (Figure S3), and this phenomenon has been observed previously with global iPLA_2_β-KO mice fed RD compared to controls [23]. In contrast, 6-month-old HFD-fed MØ-iPLA_2_β-KO male mice exhibited superior insulin sensitivity compared to floxed-iPLA_2_β control mice (Figure S3), which may reflect less HFD-induced deterioration in insulin sensitivity for 6-month-old HFD-fed MØ-iPLA_2_β-KO male mice than that which occurred for floxed-iPLA_2_β control male mice. Discrepant ITT findings between male and female global iPLA_2_β-KO mice and their responses to dietary stress have also been observed previously [23,24] and are commonplace in animal models of perturbed glucose homeostasis.

## 4. Discussion

Our previous studies with global iPLA_2_β-knockout mice indicated that disturbances in glucose homeostasis, which included glucose intolerance and impaired insulin secretion by pancreatic islet β-cells, occurred as a consequence of iPLA_2_β gene disruption [23,24]. Although iPLA_2_β and its products might participate in multiple events in GSIS from β-cells, one of them is to amplify depolarization-induced [Ca^2+^] entry into the β-cell. In addition, iPLA_2_β appears to confer protection against lipid injury to β-cells that may reflect iPLA_2_β participation in the repair of oxidative damage to β-cell mitochondrial membranes that occurs in the context of lipid toxicity [24,25]. Loss of these actions of iPLA_2_β in β-cells provides a plausible explanation for the impaired glucose tolerance in global iPLA_2_β-null mice, the reduced insulin secretory response to glucose of islets isolated from global iPLA_2_β-null mice compared to their wild-type littermates, and the exaggerated deterioration in glucose tolerance for global iPLA_2_β-null mice fed a HFD compared to wild-type controls [23,24].

More puzzling is the superior sensitivity to insulin of global iPLA_2_β-null mice compared to wild-type controls in insulin tolerance tests [24], implying that iPLA_2_β gene deletion has opposing effects on insulin secretion from β-cells and the insulin responsiveness of peripheral tissues, including skeletal muscle. One potential explanation for these findings is that iPLA_2_β deficiency has distinct effects in different cells. A loss of iPLA_2_β in β-cells would reasonably be expected to impair insulin secretion and to amplify lipid-induced β-cell injury, but a loss of iPLA_2_β activity is some other cell type might be responsible for the amelioration of lipid-induced deterioration in insulin sensitivity of global iPLA_2_β-null mice compared to wild-type controls. Candidates include cells of the monocyte/macrophage lineage. It is postulated that in the setting of lipid stress, the migration of blood monocytes into extravascular sites, including adipose tissue, and their differentiation into tissue macrophages results in the elaboration of cytokines, including IL-1β, IL-6 and TNFα, which impair insulin sensitivity. Because iPLA_2_β-derived 2-lysophosphatidic acid (LPA) appears to be required for monocyte migration in response to the cytokine Monocyte Chemoattractant-1 (MCP-1) [29,37,38,39], we postulated that iPLA_2_β-deficiency in cells of the monocyte/macrophage lineage might confer protection against the HFD-induced deterioration of insulin sensitivity because of a failure of iPLA_2_β-null monocytes to migrate into peripheral tissues and differentiate into macrophages.

We therefore prepared mice that are selectively deficient in iPLA_2_β in β-cells or cells of the monocyte/macrophage lineage. Mice with “floxed” iPLA_2_β genes were prepared using embryonic stem cells from the EUCOMM consortium. These cells had incorporated a targeting construct by homologous recombination that replaced the wild-type iPLA_2_β gene. In this construct, LoxP sites recognized by Cre recombinase [41,42] flanked critical iPLA_2_β gene exons. Mice with the “floxed” iPLA_2_β genes were then mated with mice that express Cre recombinase under the control of promoters that are expressed in only a restricted set of cells. In cells that express Cre, the action of the recombinase removes the “floxed” segment of the iPLA_2_β gene-targeting construct, and transcription of this modified gene yields a truncated mRNA that is eliminated by a nonsense mediated decay and cannot lead to production of an active iPLA_2_β protein. In this way, mice were produced that are selectively iPLA_2_β-deficient in specifically targeted populations of cells that express Cre recombinase.

To direct Cre expression in β-cells, RIP-Cre mice were used that express Cre under control of the Rat Insulin 2 Promoter, which has been demonstrated to be appropriate for β-cell-specific gene deletion when used in mice on a pure C57BL/6J background [49], as is the case in this study, although anomalies may be encountered on other genetic backgrounds [53]. The use of β-cell-targeting promoters has been reviewed [60], and the use of RIP-Cre remains a popular means of directing β-cell-restricted gene deletion [46,50,61,62,63,64,65,66,67,68,69]. The LysM promoter is expressed in all myeloid cells, and LysM-Cre is widely used to study conditional macrophage-myeloid cell gene deletions [52,70,71,72,73,74,75,76]. 

Our expectations for the behavior of these conditional iPLA_2_β-KO mouse lines was that the β-cell-iPLA_2_β-KO mice would exhibit glucose intolerance, impaired insulin secretion, and increased deterioration in glucose tolerance compared to control mice in response to a HFD, and our observations largely conformed to these expectations. Expectations for the MØ-iPLA_2_β-KO mice were that insulin secretion and glucose tolerance would be preserved, that deterioration in glucose tolerance induced by a HFD would be blunted compared to floxed-iPLA_2_β control mice, and that insulin sensitivity as measured in insulin tolerance tests (ITTs) would be impaired less in HFD-fed MØ-iPLA_2_β-KO compared to floxed-iPLA_2_β control mice, and our observations also largely conformed to these expectations.

An unexpected finding is that HFD-fed β-cell-iPLA_2_β-KO mice also had superior insulin sensitivity compared to floxed-iPLA_2_β control mice as assessed with ITTs. The explanation for this finding has not been established, but of possible relevance is the phenomenon of selective insulin resistance first proposed by McGarry [75] and subsequently discussed by Brown and Goldstein [76] and others [77,78]. McGarry proposed that hyperinsulinemia itself elicits insulin resistance in peripheral tissues [75]. During the evolution of T2D (type 2 diabetes), resistance develops to the effect of insulin to decrease hepatic gluconeogenesis but such resistance does not develop the insulin-stimulated lipogenesis of fatty acids and triacylglycerols (TAGs), which is now known to involve the activation of the transcription factor SREBP-1c [76,77,78]. In fact, hepatic lipogenesis and Very Low Density Lipoprotein (VLDL) secretion are amplified by the hyperinsulinemia that results from the resistance of peripheral tissues to the action of insulin, and fatty acids derived from VLDL TAG exacerbate the insulin-resistant state in muscle and adipose tissue [75,76,77,78,79]. These events result in the classic T2DM triad of hyperglycemia, hyperinsulinemia, and hypertriglyceridemia [75,76]. McGarry suggested that VLDL TAG play a toxic role in the evolution of T2D by their deposition in muscle, where they enhance insulin resistance, and in liver, where they can produce non-alcoholic steatohepatitis (NASH). The term lipotoxicity is used to describe the detrimental effects of accumulation of TAG and other lipids in various tissues [80], and ceramides are thought to be among the lipid mediators that contribute to insulin resistance [79].

If hyperinsulinemia is involved in inducing peripheral tissue insulin resistance, then diabetes in a mouse model caused solely by a β-cell secretory defect might fail to generate sufficiently high levels of hyperinsulinemia to drive development of insulin resistance. Compared to a control population of mice that do develop sufficient hyperinsulinemia, mice with the pure secretory defect might thus exhibit superior insulin sensitivity in insulin tolerance tests. It is of interest in this regard that knockout mice with defective K_ATP_ channel activity also have impaired insulin secretion from β-cells but enhanced insulin sensitivity of peripheral tissues in insulin tolerance tests [81], which the authors postulated might be mediated by altered extracellular hormonal or neuronal signals perturbed by disruption of K_ATP_ channels [82]. In the context of the discussion in this and the preceding paragraph, one such altered extracellular hormonal signal might be the blood insulin concentration itself.

The possibility that the insulin secretory defect of global iPLA_2_β-knockout mice and of β-cell-iPLA_2_β-KO mice prevents the hyperinsulinemia-driven deposition of toxic lipids in tissues that otherwise occurs in mice subjected to a HFD is of interest in the context of observations that hepatic steatosis fails to develop in global iPLA_2_β-knockout mice subjected to a HFD, although it does occur in control mice [83]. Gene deletion of iPLA_2_β also greatly attenuates hepatic steatosis that otherwise occurs in the *ob/ob* mouse genetic model [84]. In addition, ceramides such as those that accumulate in tissues that develop insulin resistance [79,80], also accumulate in β-cells subjected to ER stress in an iPLA_2_β-dependent process that involves activation of SREBP-1 [85,86,87,88], which is of interest in the context of involvement of SREBP-1 activation in the pathogenesis of hyperinsulinemia-driven amplification of hepatic lipogenesis in the evolution of T2D [76,77,78].

In contrast to the unexpected resistance to HFD-induced deterioration in insulin-responsivity of β-cell-iPLA_2_β-KO mice discussed above, it was expected that HFD-fed MØ-iPLA_2_β-KO mice would exhibit superior insulin sensitivity compared to HFD-fed floxed-iPLA_2_β control mice as assessed with ITTs. This expectation was based on the postulates that HFD-induced deterioration in insulin sensitivity involves migration of blood monocytes into extravascular sites, such as adipose tissue, where they differentiate into macrophages and produce cytokines, including IL-β, IL-6, and TNFα, that impair insulin sensitivity in a process that involves the tissue elaboration of the cytokine Monocyte Chemoattractant-1 (MCP1) and its interaction with the CCR2 receptor on monocytes to induce their migration into tissues [31,32,33,34,35,36]. Genetic and pharmacologic evidence indicates that the monocyte chemotactic response to MCP-1 requires the action of iPLA_2_β to produce the lipid mediator 2-lysophosphatidic acid (LPA) [37,38,39]. Macrophages [29,30] and other cells [89] from our global iPLA_2_β knockout mice have been demonstrated to have impaired production of LPA in response to stimuli that induce robust LPA production in wild-type cells. 

Moreover, macrophages from our global iPLA_2_β knockout mice have impaired migratory responses to MCP-1 in a mouse model of diet-induced glucose intolerance and atherogenesis, and these migratory responses are restored by provision of exogenous LPA [29]. The accumulation of macrophages in atherosclerotic lesions in this mouse model of diabetic stress is also associated with increased lesion content of iPLA_2_β immunoreactive protein and enzymatic activity [29]. Migratory responses of vascular smooth muscle cells (VSMCs) in a model of vascular injury are also greatly reduced in our global iPLA_2_β-null mice compared to wild-type mice [90]. The activation state of iPLA_2_β-null macrophages from our global iPLA_2_β knockout mice is also shifted toward an M2 anti-inflammatory phenotype compared to the inflammatory M1 phenotype exhibited by wild-type macrophages, and this is associated with reduced TNFα production by the iPLA_2_β-null macrophages [30,91,92,93], which suggests that in addition to participating in monocyte migration into extravascular tissues, iPLA_2_β may also be involved in their differentiation into pro-inflammatory macrophages that elaborate cytokines that impair insulin sensitivity. The pharmacologic inhibition of iPLA_2_β also ameliorates leukocyte infiltration into pancreatic islets and the onset of diabetes in NOD (non-obese diabetic) mice [92,93], which suggests that iPLA_2_β may participate in the evolution of T1D in addition to that of T2DM.

iPLA_2_β is in fact involved in regulating several fundamental aspects of monocyte/macrophage biology in addition to those discussed above, including the remodeling the fatty acid composition of macrophage phospholipids [94,95], selection of macrophage phospholipid pools from which to mobilize fatty acids upon cellular stimulation [27,96,97], governing the proliferation state of macrophage precursor cells [97], regulating macrophage apoptosis in the context of lipid stress [98], regulating the rate of transcription of the macrophage inducible nitric oxide synthase (iNOS) gene [26], and promoting macrophage adhesion and spreading in response to the engagement of the class A scavenger receptor [99]. Development of the monocyte/macrophage-selective conditional iPLA_2_β-knockout mice described here may prove to be useful in further characterizing these aspects of monocyte/macrophage biology in models of diabetes, atherosclerosis, and other pathophysiologic states. 

In addition, preparation of the mouse line with floxed-iPLA_2_β genes described here may prove to be useful in the development of conditional knockout mouse lines with selective deletion of iPLA_2_β in other cell lines that might participate in the development of T2DM, including skeletal muscle cells, adipocytes, and hepatocytes. All of these cell types are involved in glucose homeostasis in T2D, and all express iPLA_2_β [83,84,100,101], which is involved in regulating fatty acid oxidation in skeletal muscle [100], differentiation of adipoctyes [101], and hepatic lipid synthesis [83,84]. Conditional knockouts in skeletal muscle can be prepared from our iPLA_2_β-floxed mice with mice that express Cre under the control of the promoter for muscle creatine kinase (MCK) [102], conditional knockouts in adipocytes can be prepared with our floxed mice and mice that express Cre under control of the promoter for adipocyte-specific fatty acid binding protein (aP2) [103]. Conditional knockouts in hepatocytes can be prepared with our floxed mice and mice that express Cre under control of the promoter for rat albumin [54]. Such lines would permit further characterization of the mechanism of resistance of the global iPLA_2_β knockout to HFD-induced deterioration in insulin sensitivity [24].

## 5. Conclusions

Cre-lox technology was used to produce mice with selective iPLA_2_β deficiency in cells of myelomonocytic lineage, including macrophages (MØ-iPLA_2_β-KO), or in insulin-secreting β-cells (β-Cell-iPLA_2_β-KO), respectively. MØ-iPLA_2_β-KO mice exhibited normal glucose tolerance when fed standard chow and better glucose tolerance than floxed-iPLA_2_β control mice after consuming a high-fat diet (HFD). MØ-iPLA_2_β-KO mice exhibited normal GSIS in vivo and from isolated islets ex vivo compared to floxed-iPLA_2_β controls. Male MØ-iPLA_2_β-KO mice exhibited enhanced insulin responsivity vs. controls after a prolonged HFD. β-Cell-iPLA_2_β-KO mice exhibited impaired glucose tolerance when fed standard chow, and glucose tolerance deteriorated further after introduced to a HFD. β-Cell-iPLA_2_β-KO mice exhibited impaired GSIS in vivo and from isolated islets ex vivo vs. controls, and male β-cell-iPLA_2_β-KO mice also exhibited enhanced insulin responsivity compared to controls after of introduction of the HFD. These findings suggest that MØ iPLA_2_β participates in HFD-induced deterioration in glucose tolerance, and that this mainly reflects an effect on insulin responsivity rather than on insulin secretion. In contrast, β-cell iPLA_2_β plays a role in GSIS and also appears to confer some protection against deterioration in β-cell function induced by a HFD.

## Supplementary Materials

The following are available online at https://www.mdpi.com/2218-273X/10/10/1455/s1, Figure S1: In Vivo Insulin Secretion For iPLA_2_β Conditional Knockout and Floxed-iPLA_2_β Control Mice Fed a Regular or High-Fat Diet, Figure S2: Insulin Tolerance Tests of 6-month-old Female Conditional iPLA_2_β-Knockout Mice and Floxed-iPLA_2_β Controls Fed a Regular or High-Fat Diet (HFD), Figure S3: Insulin Tolerance Tests of 6-month-old Male Conditional iPLA_2_β-Knockout Mice and Floxed-iPLA_2_β Controls Fed a Regular or High-Fat Diet (HFD). Table S1: Glucose Tolerance Tests for Female and Male Mice Aged 3 or 6 months with Genotypes Floxed-iPLA_2_β, β-Cell-iPLA_2_β-KO, or MØ-iPLA_2_β-KO Fed a Regular or High-Fat Diet.
