# Supplementary material for: Metabolic Effects of Selective Deletion of Group VIA Phospholipase A2 from Macrophages or Pancreatic Islet Beta-Cells
Source: Biomolecules. 2020 Oct 17;10(10):1455. doi: 10.3390/biom10101455 (PMC7602969; doi:10.3390/biom10101455)
Supplement: Supplementary file 1 [file biomolecules-10-01455-s001.pdf]

## Supplementary Materials

**Table 1.** Glucose tolerance tests for female and male mice aged 3 or 6 months with genotypes floxed-iPLA$_2$β, β-Cell-iPLA$_2$β-KO, or MØ-iPLA$_2$β-KO fed a regular or high-fat diet. Glucose tolerance tests (GTTs) were performed as in Figure 2, and the Areas Under the Curve (AUC) were computed as in Figure 3 for female (denoted "F") and male (denoted "M") floxed-iPLA$_2$β control mice, MØ-iPLA$_2$β-KO mice, and β-cell-iPLA$_2$β-KO mice aged 3 months or 6 months that had been fed a RD until age 8 weeks and then either RD or a HFD until age 3 months or 6 months. Values are displayed as means ± SEM (n = 6 to 25, as specified for each entry in the Table). Tabulated significance values pertain to the comparison between genotypes for the condition in question.

| *Entry* | *Age (mos.)* | *Gender* | *Genotype* | *Diet* | *GTT AUC* | *SEM* | *n* | *p value* |
|---|---|---|---|---|---|---|---|---|
| 1 | 3 | F | Floxed-iPLA$_2$β | Regular | 269 | 13 | 19 | |
| 2 | 3 | F | MØ-iPLA$_2$β-KO | Regular | 231 | 10 | 19 | ***0.028*** |
| 3 | 3 | F | Floxed-iPLA$_2$β | High Fat | 374 | 19 | 18 | |
| 4 | 3 | F | MØ-iPLA$_2$β-KO | High Fat | 352 | 18 | 18 | 0.414 |
| 5 | 3 | F | Floxed-iPLA$_2$β | Regular | 280 | 19 | 13 | |
| 6 | 3 | F | β-Cell-iPLA$_2$β-KO | Regular | 367 | 26 | 8 | ***0.008*** |
| 7 | 3 | F | Floxed-iPLA$_2$β | High Fat | 435 | 23 | 6 | |
| 8 | 3 | F | β-Cell-iPLA$_2$β-KO | High Fat | 514 | 34 | 6 | 0.083 |
| 9 | 3 | M | Floxed-iPLA$_2$β | Regular | 321 | 28 | 20 | |
| 10 | 3 | M | MØ-iPLA$_2$β-KO | Regular | 327 | 23 | 15 | 0.881 |
| 11 | 3 | M | Floxed-iPLA$_2$β | High Fat | 509 | 22 | 13 | |
| 12 | 3 | M | MØ-iPLA$_2$β-KO | High Fat | 508 | 28 | 16 | 0.988 |
| 13 | 3 | M | Floxed-iPLA$_2$β | Regular | 389 | 25 | 12 | |
| 14 | 3 | M | β-Cell-iPLA$_2$β-KO | Regular | 475 | 39 | 13 | 0.081 |
| 15 | 3 | M | Floxed-iPLA$_2$β | High Fat | 566 | 21 | 12 | |
| 16 | 3 | M | β-Cell-iPLA$_2$β-KO | High Fat | 583 | 42 | 12 | 0.72 |
| 17 | 6 | F | Floxed-iPLA$_2$β | Regular | 265 | 10 | 24 | |
| 18 | 6 | F | MØ-iPLA$_2$β-KO | Regular | 250 | 10 | 23 | 0.278 |
| 19 | 6 | F | Floxed-iPLA$_2$β | High Fat | 485 | 25 | 24 | |
| 20 | 6 | F | MØ-iPLA$_2$β-KO | High Fat | 414 | 22 | 24 | ***0.038*** |
| 21 | 6 | F | Floxed-iPLA$_2$β | Regular | 317 | 14 | 21 | |
| 22 | 6 | F | β-Cell-iPLA$_2$β-KO | Regular | 454 | 22 | 20 | ***4.14E-06*** |
| 23 | 6 | F | Floxed-iPLA$_2$β | High Fat | 549 | 42 | 12 | |
| 24 | 6 | F | β-Cell-iPLA$_2$β-KO | High Fat | 838 | 28 | 12 | ***1.45E-05*** |
| 25 | 6 | M | Floxed-iPLA$_2$β | Regular | 340 | 24 | 19 | |
| 26 | 6 | M | MØ-iPLA$_2$β-KO | Regular | 396 | 20 | 20 | 0.079 |
| 27 | 6 | M | Floxed-iPLA$_2$β | High Fat | 728 | 27 | 25 | |
| 28 | 6 | M | MØ-iPLA$_2$β-KO | High Fat | 645 | 31 | 25 | ***0.049*** |
| 29 | 6 | M | Floxed-iPLA$_2$β | Regular | 435 | 46 | 5 | |
| 30 | 6 | M | β-Cell-iPLA$_2$β-KO | Regular | 621 | 64 | 4 | ***0.0085*** |
| 31 | 6 | M | Floxed-iPLA$_2$β | High Fat | 756 | 37 | 12 | |
| 32 | 6 | M | β-Cell-iPLA$_2$β-KO | High Fat | 870 | 21 | 12 | ***0.014*** |

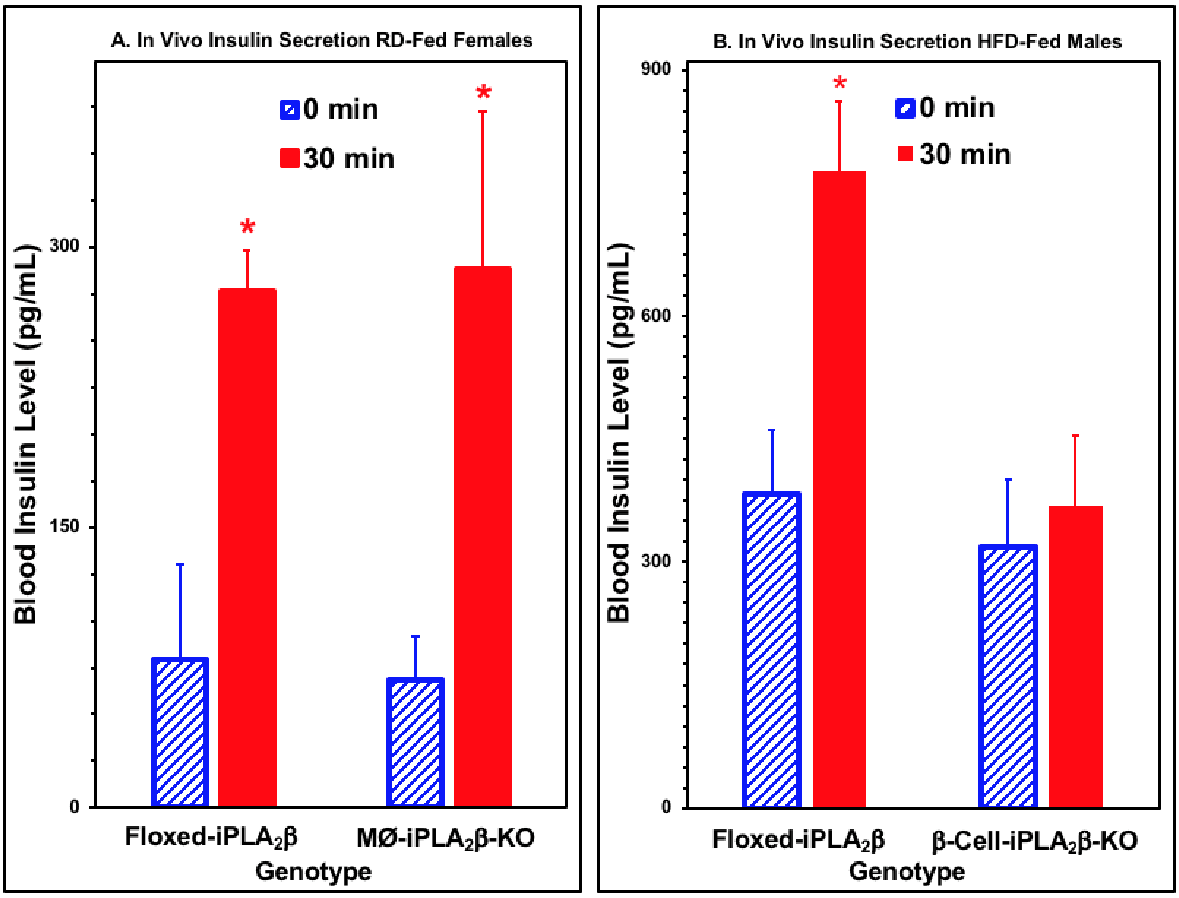


**Figure S1.** In vivo insulin secretion for iPLA$_2$β conditional knockout and floxed- iPLA$_2$β control mice fed a regular or high-fat diet. After an overnight fast, baseline blood samples were obtained from the saphenous vein of female (**A**) or male (**B**) MØ-iPLA$_2$β-KO mice (**A**), β-cell-iPLA$_2$β-KO mice (**B**), or floxed-iPLA$_2$β control mice 6 months of age that had been fed a regular diet (RD, **A**) or high-fat diet (HFD, **B**) after 8 weeks of age. D-glucose (3 mg/kg body weight) was administered by intraperitoneal injection, and a blood sample was obtained 30 min thereafter. The insulin content of the baseline (cross-hatched bars) and 30 min (solid bars) samples was then measured by enzyme-linked immunosorbent assay, as described [23,24,57]. Displayed values represent mean ± SEM. An asterisk (*) denotes $p < 0.05$ for the comparison between the time 0 and 30 min values (n = 5 to 7).

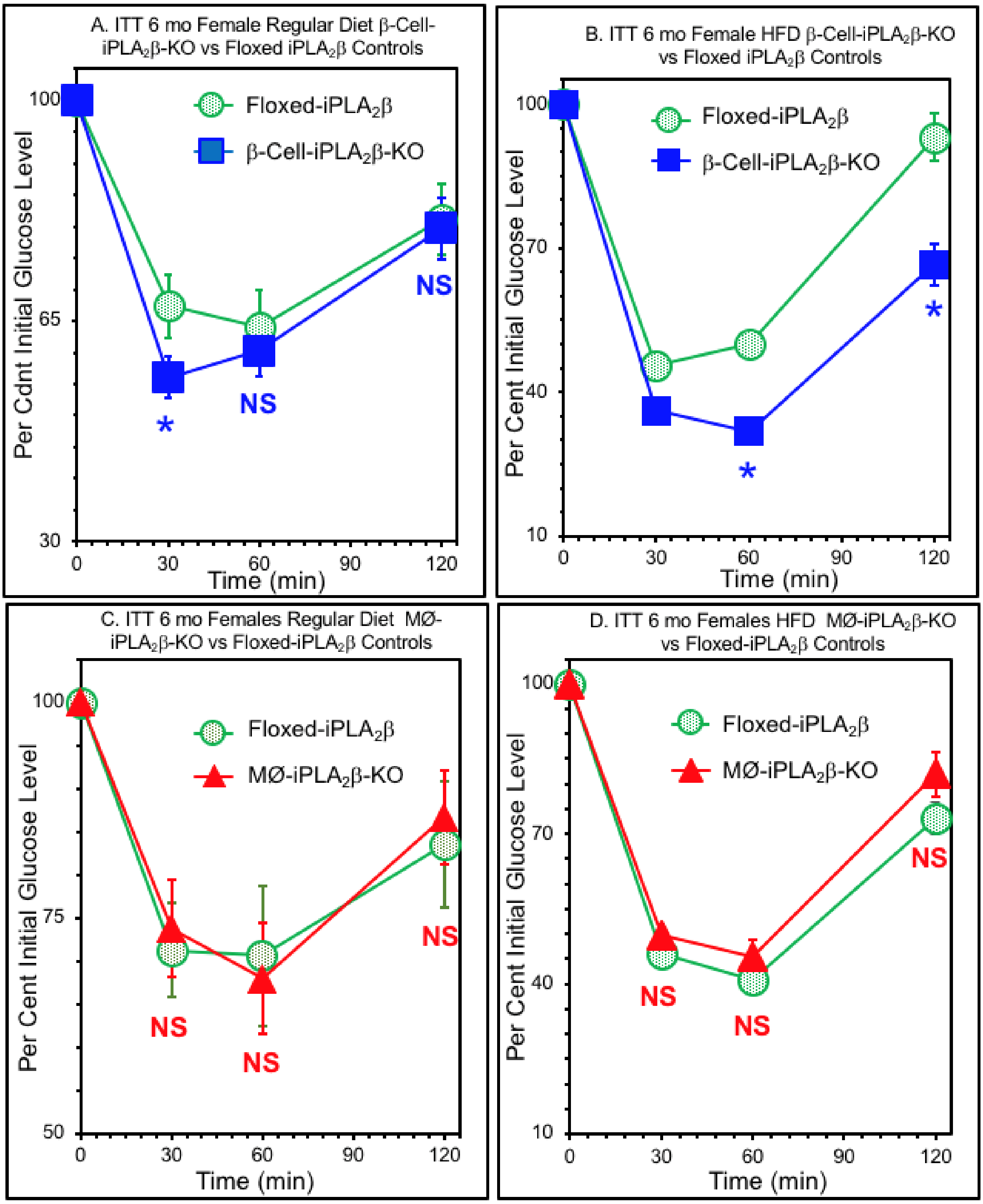


**Figure S2.** Insulin tolerance tests of 6-month-old female conditional iPLA2β-knockout mice and floxed-iPLA2β controls fed a regular or high-fat diet (HFD). Female floxed-iPLA2β control mice (circles), β-cell-iPLA2β-KO mice (squares, **A,B**) mice, or MØ-iPLA2β-KO mice (triangles, **C,D**) were fed a RD after weaning until they were 8 weeks of age and were then fed a HFD until 6 months of age. Insulin tolerance tests were then performed in mice with free access to water and chow until human regular insulin (0.75 U/kg; Lilly, Indianapolis, IN) was administered by intraperitoneal injection. Blood specimens were collected at 0, 30, 60, and 120 min thereafter for glucose concentration measurements, which were expressed as a percentage of the time zero blood glucose concentration, as described in [23,24,57]. Displayed values represent mean ± SEM (n = 13 to 26). An asterisk (*) denotes $p < 0.05$ for the comparison between genotypes.

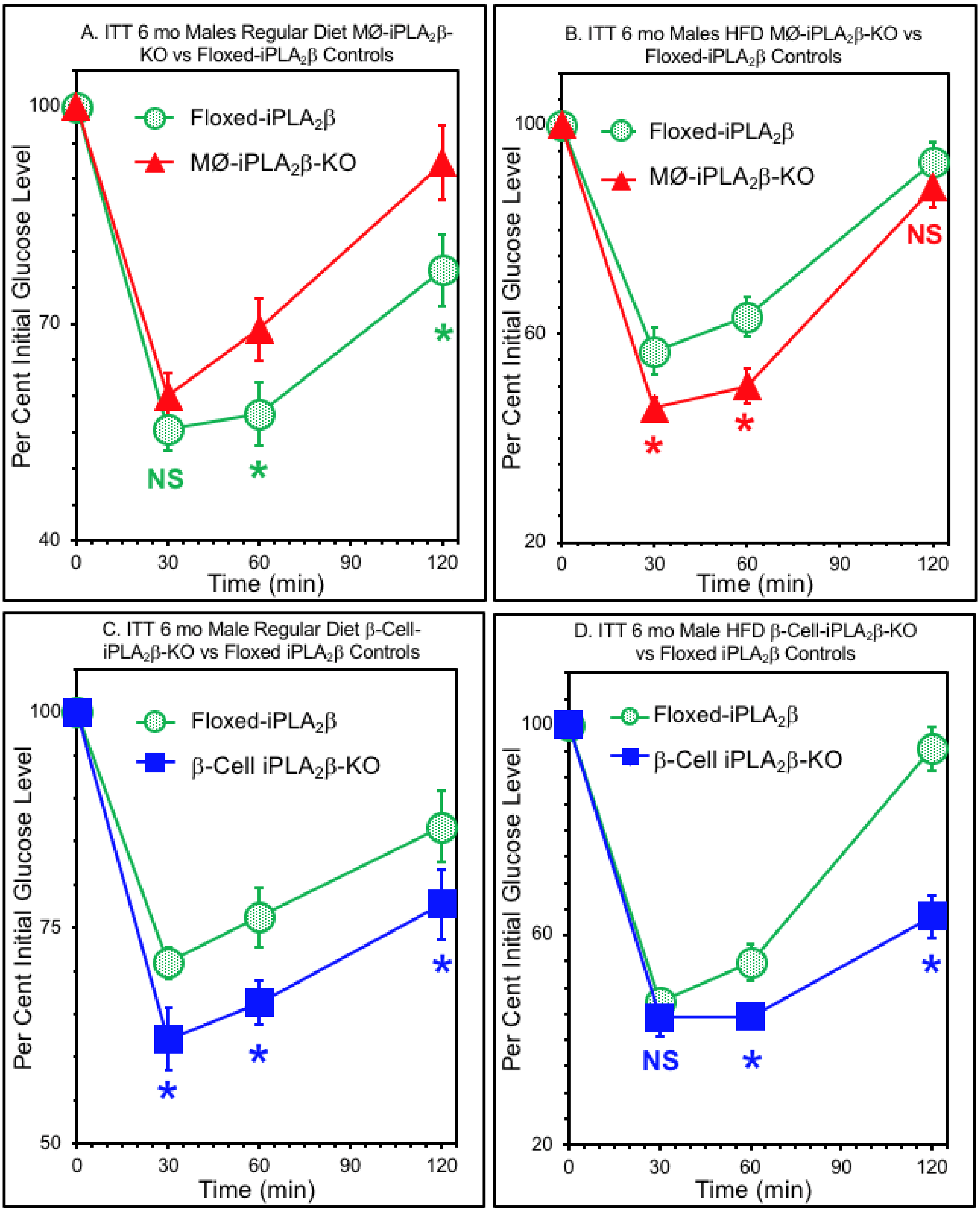


**Figure S3.** Insulin tolerance tests of 6-month-old male conditional $iPLA_2\beta$-knockout mice and floxed-$iPLA_2\beta$ Controls Fed a Regular or High-Fat Diet (HFD). Male floxed-$iPLA_2\beta$ control mice (circles), MØ-$iPLA_2\beta$-KO mice (triangles, **A,B**), or β-cell-$iPLA_2\beta$-KO mice (squares, **C,D**) mice were fed a RD after weaning until they were 8 weeks of age and were then fed a HFD until 6 months of age. Insulin tolerance tests were then performed in mice with free access to water and chow until human regular insulin (0.75 U/kg body weight; Lilly, Indianapolis, IN) was administered by intraperitoneal injection. Blood specimens were collected at 0, 30, 60, and 120 min thereafter for glucose concentration measurements, which were expressed as a percentage of the time zero blood glucose concentration, as described in [23,24,57]. Displayed values represent mean ± SEM (n = 13 to 26). An asterisk (*) denotes $p < 0.05$ for the comparison between genotypes.
